# Stromal cells unifying the pathology of acute and chronic skeletal muscle injury – clue for novel biomaterials

**DOI:** 10.3389/fbioe.2025.1684310

**Published:** 2025-10-03

**Authors:** Xianglong Liu

**Affiliations:** Department of Chemical and Biomolecular Engineering, National University of Singapore, Singapore, Singapore

**Keywords:** skeletal muscle, regeneration, degeneration, biomaterials, stromal cells

Skeletal muscle degeneration manifests through two clinically significant yet mechanistically distinct pathological processes - muscle fatty infiltration (MFI) and fibrosis. These conditions represent major obstacles in the management of age-related muscle wasting, disuse atrophy, and post-traumatic recovery (Cuijpers et al., 2025; Jang and Choi, 2025; Ge et al., 2025; Guo et al., 2025). Intramuscular fat accumulation progressively replaces functional muscle tissue, leading to metabolic dysfunction and weakness, while excessive fibrotic deposition disrupts normal muscle architecture and impairs regenerative capacity (Yu et al., 2025; Farahat et al., 2025). Recent advances in single-cell technologies have begun to unravel the complex cellular dynamics underlying these conditions, revealing unexpected roles for ferroptosis regulation in fibro/adipogenic progenitors (FAPs) during chronic fatty infiltration and macrophage plasticity in acute fibrotic responses, which opens new avenues for targeted interventions in muscle degenerative disorders (Vitaliti et al., 2024; Uapinyoyin et al., 2023).

The study by Sun’ team provides compelling evidence for a previously unrecognized role of ferroptosis suppression in disuse-induced MFI (Tan et al., 2025). Their comprehensive single-cell analysis of immobilized murine muscle uncovered a remarkable subpopulation of FAPs that not only escaped ferroptosis cell death but actively upregulated genes involved in ferroptosis resistance. This finding challenges the conventional view of ferroptosis as a uniformly detrimental process in muscle degeneration. Through elegant *in vitro* experiments, the researchers demonstrated that pharmacological inhibition of ferroptosis using ferrostatin-1 significantly enhanced the adipogenic potential of both primary FAPs and muscle-derived fibroblasts. These results suggest that the metabolic rewiring of FAPs to resist ferroptosis creates permissive conditions for their pathological differentiation into fat-depositing cells. The study’s clinical relevance is particularly noteworthy, as it identifies ferroptosis modulation as a potential therapeutic strategy to prevent MFI in conditions ranging from sarcopenia to prolonged immobilization.

In contrast to the chronic process of fatty infiltration, acute muscle injury often leads to fibrotic scarring and even bulk lesion, where this team have made groundbreaking discoveries regarding macrophage plasticity. Their work represents the first definitive evidence of macrophage-to-myofibroblast transition (MMT) occurring in skeletal muscle tissue (Qi et al., 2024). By combining sophisticated single-cell transcriptomic approaches with carefully timed *in vivo* sampling, the researchers captured the dynamic conversion of anti-inflammatory macrophages into collagen-producing myofibroblasts during the critical window of muscle repair. Making a step further, Luo et al. prepared a bioactive hydrogel for regulating macrophage dynamics to promote muscle regeneration after acute trauma (Luo et al., 2025). These findings are particularly exciting as they have unveiled the multifaceted role of macrophages in skeletal muscle regeneration and identified a therapy for skeletal muscle regeneration by inhibiting maladaptive MMT while preserving the beneficial roles of macrophages. This is of particular importance as the promotion of skeletal muscle neogenesis can also serve as a foundation for alleviating muscle atrophy induced by aging or disuse.

The convergence of these serial studies highlights the remarkable plasticity of muscle stromal cells in response to different pathological stimuli. While Tan et al. demonstrate how FAPs adapt their metabolic program to resist ferroptosis and promote fatty degeneration, Qi et al. reveal the unexpected trans-differentiation potential of macrophages in fibrotic scarring. Together, these findings paint a more complete picture of muscle degeneration, where distinct cellular mechanisms dominate different disease phases and contexts. Important questions emerge regarding potential crosstalk between these pathways - could ferroptosis-resistant FAPs create a microenvironment that favors MMT? Might macrophage-derived factors influence the adipogenic potential of resident FAPs? Addressing these questions will be crucial for developing comprehensive treatment strategies that account for the complex cellular interactions in degenerating muscle.

The findings of Sun et al. serve as a basis for tissue engineering in skeletal muscle repair. Previous researches usually focus on the regeneration or hypertrophy of myofiber. However, the formation of multi-nuclei myofiber from mono-nuclei stem cells is a complex process requiring different stimuli in a sequential manner, making it difficult to fabricate appropriate biomaterials. Sun’s team indicates that the fine modulation of stromal cells, including but not limited to FAPs and macrophages, can also be a potential way to regulate skeletal muscle regeneration.

As the understanding of muscle pathophysiology continues to evolve, studies like these that uncover novel cellular behaviors and regulatory mechanisms will be instrumental in developing more effective treatments. The application of advanced single-cell technologies, as demonstrated in both papers, promises to reveal further complexity in muscle degenerative processes and identify additional therapeutic targets. Ultimately, this growing knowledge base should lead to more precise interventions that can maintain muscle quality and function across diverse clinical contexts.
